# Comparing the anorexigenic effects and mechanisms of gut-derived GLP-1 and its receptor agonists: insights into incretin-based therapies for obesity

**DOI:** 10.1007/s13340-025-00819-9

**Published:** 2025-05-20

**Authors:** Yuta Masuda, Kento Ohbayashi, Yusaku Iwasaki

**Affiliations:** Laboratory of Animal Science, Graduate School of Life and Environmental Sciences, Kyoto Prefectural University, 1-5 Hangi-cho, Shimogamo, Sakyo-ku, Kyoto, 606-8522 Japan

**Keywords:** Glucagon like peptide-1 (GLP-1), GLP-1 receptor agonist, Glucose-dependent insulinotropic polypeptide (GIP), Obesity, Food intake, Vagal afferent nerves

## Abstract

Obesity continues to increase worldwide. The primary cause of obesity is overeating, but the development of pharmacological treatments for obesity related to overeating has taken longer than expected. Recently, agonists of glucagon-like peptide-1 (GLP-1) receptor, designed based on the gut hormone GLP-1, have been developed as anti-obesity drugs and have demonstrated remarkable efficacy in treating both obesity and diabetes. Meanwhile, recent research using factors that promote GLP-1 secretion has highlighted the significance of endogenous GLP-1 function. This review provides an overview of the anorexigenic effects, adverse effects, and their underlying mechanisms of GLP-1 receptor agonists and endogenous gut-derived GLP-1. Furthermore, it discusses the potential anti-obesity effects of dual agonists targeting both the glucose-dependent insulinotropic polypeptide (GIP) receptor and the GLP-1 receptor, which have gained attention in recent years. Finally, we compare the beneficial effects of GLP-1 receptor agonists and meal-induced gut GLP-1 secretion on overeating-induced obesity and discuss how combining these approaches may complement each other’s limitations and serve as a promising long-term strategy for preventing and treating obesity.

## Introduction

The number of individuals with obesity continues to increase globally. According to a report by the World Obesity Federation, it is predicted that by 2023, one in four to five people will be classified as obese. Obesity is a well-established major risk factor for metabolic disorders including diabetes. The primary cause of obesity is excessive food intake. Historically, the development of appetite-suppressing drugs has focused on targeting receptors or transporters of neurotransmitters and neuromodulators released by neurons involved in central feeding regulation. However, many of these drugs were associated with unexpected adverse effects, such as nausea, aversion, valvular heart disease, and even suicidal tendencies, leading to the discontinuation of most of their development. In contrast, drugs modeled after gastrointestinal hormones, such as glucagon-like peptide-1 (GLP-1), have been shown to exhibit fewer side effects while demonstrating high efficacy in obesity treatment.

This review outlines the anti-obesity effects and mechanisms of action of emerging anti-obesity drugs, including GLP-1 receptor agonists and dual GIP/GLP-1 receptor agonists. Furthermore, it provides an overview of the anti-obesity and anti-diabetic effects mediated by the enhanced endogenous secretion of GLP-1 and their underlying mechanisms.

## Anorexigenic effects and underlying mechanisms of GLP-1 receptor agonists

### Overview of GLP-1 receptor agonists

GLP-1 receptor agonists (GLP-1RAs) are drugs developed for the treatment of type 2 diabetes and obesity. They include exenatide, from the Gila monster (*Heloderma suspectum*), and human GLP-1 analogs, such as liraglutide and semaglutide. Based on their duration of action, they are classified as short-acting or long-acting. GLP-1RAs are effective in improving hyperglycemia and obesity by regulating glycemic control, suppressing excessive food intake, and reducing body weight. Exenatide was the first GLP-1 receptor agonist approved by the FDA for the treatment of type 2 diabetes in 2005. Similarly, liraglutide and semaglutide were approved in the United States as anti-obesity medications in 2014 and 2021, respectively. Currently, these drugs are used in many countries including Japan.

### Mechanisms underlying the anorexigenic effects of GLP-1 receptor agonists

The anorexigenic effects of GLP-1RAs are primarily mediated by the activation of GLP-1Rs expressed in the brain. GLP-1Rs are not only widely expressed in the central nervous system but also in peripheral nerves such as vagal afferents. When the GLP-1R was knocked out specifically in all neurons using nestin-driven knockout mice (nestin^Cre/+^; Glp1r^flox/flox^), the anorexigenic effect of liraglutide was markedly attenuated [1]. In contrast, liraglutide still reduced food intake in mice with GLP-1R knockout limited to autonomic neurons including vagal afferent neurons (phox2b-Cre; Glp1r^flox/flox^) [1]. Sub-chronic administration of liraglutide in rats significantly suppressed weight gain by reducing food intake, and this effect was not affected by deafferentation of subdiaphragmatic vagal afferents [2]. Notably, although the mechanism remains unclear, exenatide (exendin-4) did not activate vagal afferent nerves in either ex vivo or in vivo studies in rodents [3, 4]. These findings suggest that GLP-1RAs directly act on GLP-1Rs in the brain to suppress food intake.

**Fig. 1:**
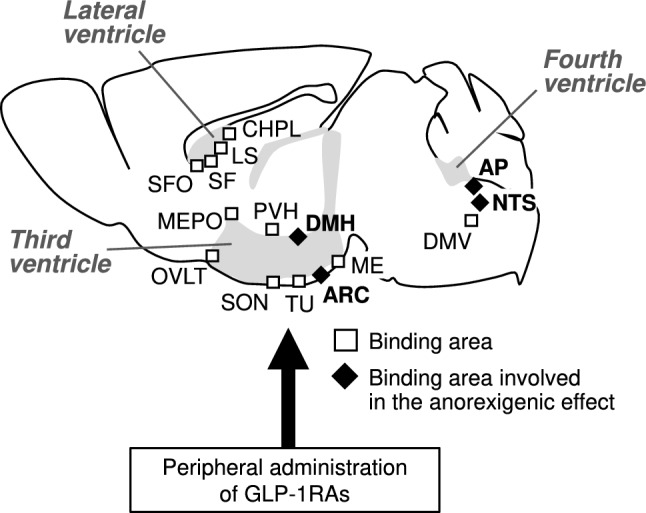
GLP-1 receptor-expressing area targeted by peripheral administration of GLP-1 receptor agonist. Summary diagram of the results from [2, 5]. GLP-1Rs are widely expressed in the brain; however, peripherally administered GLP-1RAs (fluorescently labeled liraglutide or semaglutide) bind to GLP-1Rs in the circumventricular organs. In particular, their action on GLP-1Rs expressed in the ARC and DMH of the hypothalamus and the AP and NTS of the medulla contributes to the anorexigenic effect. Opened square, binding area; filled diamond, binding area involved in the anorexigenic effect, gray area, ventricles. AP, area postrema; ARC, arcuate nucleus; CHPL, choroid plexus; DMH, dorsomedial hypothalamic nucleus; DMV, the dorsal motor nucleus of the vagus nerve; LS, lateral septum; ME, median eminence; MEPO, median preoptic nucleus; NTS, nucleus tractus solitarius; OVLT, vascular organ of the lamina terminalis; PVN, paraventricular nucleus of the hypothalamus; SF, septofimbrial nucleus; SFO, subfornical organ; SO, supraoptic nucleus; TU, tuberal nucleus

Peripherally administered fluorescently labeled liraglutide or semaglutide have been shown to bind to the GLP-1R in the brainstem, septal nucleus, and hypothalamus; these regions are part of the circumventricular organs and specific sites near the ventricles [2, 5] (Fig. 1). Regarding the mechanism by which GLP-1RAs are taken up into the brain, it has been reported that GLP-1RAs do not cross the blood-brain barrier through endothelial cells [5]. Instead, they are taken up by tanycytes, specialized glial cells lining the third ventricle, and transported into the brain including the hypothalamus and cerebrospinal fluid [5–7]. The activation of hypothalamic neurons, suppression of food intake, and weight loss induced by liraglutide were attenuated in mice with impaired transcellular transport in tanycytes or in mice with a tanycyte-specific GLP-1 receptor knockdown [7]. These results suggest that the uptake of GLP-1RAs into the brain via tanycytes is important for their anti-obesity effects.

Regarding the central mechanism of feeding regulation, proopiomelanocortin (POMC) neurons in the arcuate nucleus (ARC) of the hypothalamus are key anorexigenic neurons. Liraglutide binds to GLP-1Rs expressed in ARC POMC neurons, thereby activating them [2]. Liraglutide-induced weight loss is blunted by the local injection of a GLP-1R antagonist into the ARC [2]. This indicates that the ARC plays a key role in the anti-obesity effects of GLP-1RAs. Furthermore, recent studies have demonstrated that GLP-1RAs may exert their anorexigenic effects not only by acting on ARC POMC neurons but also in other brain regions, such as the dorsomedial hypothalamus (DMH) and the dorsal vagal complex in the hindbrain [8, 9]. Taken together, these findings indicate that GLP-1RAs regulate food intake through a complex network of central GLP-1R rather than a single pathway (Fig. 1). Further research is needed to fully understand the neural circuits underlying their feeding suppression effects.

### Clinical therapeutic effects and challenges of GLP-1 receptor agonists

The primary GLP-1RAs used worldwide as anti-obesity drugs are liraglutide (Saxenda®, Novo Nordisk) as a once-daily subcutaneous injection and semaglutide (Wegovy®, Novo Nordisk) as a once-weekly subcutaneous injection. In non-diabetic individuals with overweight or obesity, with an average BMI of approximately 38, liraglutide administered at a dose of 3.0 mg per day for 56 weeks resulted in an 8% weight loss [10]. In contrast, semaglutide at a dose of 2.4 mg per week for 68 weeks resulted in a 14.9–15.6% weight loss in non-diabetic individuals with overweight or obesity, also with an average BMI of approximately 38 [11, 12], demonstrating a stronger anti-obesity effect with semaglutide than with liraglutide. The ~15% weight loss observed with semaglutide approaches the 20–30% weight loss achieved through bariatric surgery.

The major adverse effects of GLP-1RAs are nausea and vomiting, which occur in approximately 70% of participants [13]. One study reported that 43.8% of participants who discontinued treatment did so due to nausea and vomiting, while 36.8% stopped because of gastrointestinal discomfort [14]. The side effects are caused by the activation of GLP-1Rs in the area postrema of the medulla, which sends signals to the lateral parabrachial nucleus, leading to aversion [9]. A dual agonist for GLP-1R and glucose-dependent insulinotropic polypeptide receptor (GIPR), discussed later, may attenuate these side effects through GIP receptor signaling.

Another major concern with GLP-1RAs is weight regain after stopping treatment. Studies in both animals and humans have shown that the reduced body weight nearly returns to pre-treatment levels once the medication is stopped [15]. In obese individuals without diabetes (BMI ≥ 30), administration of semaglutide (2.4 mg/week) for 68 weeks resulted in a 17.3% reduction in body weight; however, following treatment withdrawal, body weight increased, with 11.6% of the lost weight regained after 52 weeks [16]. These findings suggest that long-term or intermittent administration of GLP-1RAs is necessary for individuals with obesity to maintain a healthy weight.

GLP-1RAs not only suppress appetite but may also affect food preferences and psychological function. A study using the Control of Eating Questionnaire (CoEQ) has reported that semaglutide treatment reduced hunger, appetite, and interest in high-palatability foods while also diminishing the pleasure of eating [17]. This result suggests that GLP-1RAs may help control overeating but could also reduce eating-related satisfaction. The risk of depression associated with GLP-1RAs remains inconclusive, as studies have reported both an increased and decreased risk [18, 19]. Since obesity and diabetes are risk factors for depression, further research is needed to determine whether the observed effects are due to the anti-obesity benefits or the pharmacological action of GLP-1RAs.

Animal studies suggest that GLP-1RAs may reduce the intake of addictive substances, such as alcohol and drugs [20]. This effect is thought to involve inhibition of dopamine neuron activity in the ventral tegmental area, a key brain region in the reward system [21, 22]. These findings suggest that GLP-1RAs may have therapeutic potential beyond diabetes and obesity, particularly in the treatment of addictions to palatable foods, alcohol, and drugs.

## Anorexigenic effects of dual GIP/GLP-1 receptor agonists

### Overview of dual GIPR/GLP-1R agonists

Developing more effective anti-obesity drugs has highlighted the importance of targeting multiple signaling pathways involved in feeding regulation [23]. Based on this hypothesis, research began on dual agonists that act on two receptors with a single molecule. In 2009, the first compound demonstrating anti-obesity effects was reported, a dual agonist targeting both the GLP-1 receptor and the glucagon receptor [24]. Subsequently, a dual agonist targeting the GIP receptor, another incretin hormone, and the GLP-1 receptor was developed. In 2013, this compound was reported to have both anti-diabetic and anti-obesity effects, with its anti-obesity efficacy surpassing that of GLP-1RAs [25]. Although the GIP and GIP receptor system is known to promote energy assimilation and contribute to obesity [26], the fact that a dual GIP/GLP-1R agonist exhibited stronger anti-obesity effects than GLP-1RAs alone was unexpected.

As the first anti-obesity drug targeting multiple receptors, tirzepatide (Zepbound®, Eli Lilly), a dual GIPR/GLP-1R agonist, was approved by the FDA in 2022. Tirzepatide consists of 39 amino acids, with its N-terminal region resembling GIP and its C-terminal region similar to the GLP-1 receptor agonist exenatide [27]. A fatty acid is attached to the lysine at position 20, increasing affinity for endogenous albumin, thereby extending its half-life and allowing for sustained therapeutic effects. Regarding its binding affinity, tirzepatide shows similar affinity for the human GIP receptor as native GIP (human GIP: Ki = 0.132 nM vs. tirzepatide: Ki = 0.135 nM). In contrast, its affinity for the human GLP-1 receptor is about one-fifth that of native GLP-1 (human GLP-1: Ki = 0.907 nM vs. tirzepatide: Ki = 4.23 nM) [27]. Furthermore, tirzepatide’s binding affinity for the mouse GIP receptor is 30 to 100 times lower than for the human GIP receptor [28]. Therefore, careful consideration is required when evaluating the pharmacological effects of tirzepatide in mouse models.

### Mechanisms underlying the anorexigenic effects of dual GIPR/GLP-1R agonists

GIPR/GLP-1R dual agonists induce significantly greater weight loss than GLP-1RAs alone in both animal models and human [29, 30]. However, the exact mechanisms by which dual agonists enhance feeding suppression beyond GLP-1RAs remain unclear. To elucidate this mechanism, studies are actively investigating the effects of GIPR agonists, as well as the expression of GIP receptors in the brain and the functional analysis of receptor-expressing neurons using approaches such as chemogenetics.

Research on the role of the GIPR in feeding regulation has expanded significantly following the development of GIPR agonists. Studies using GIP-Cre mice have demonstrated that GIP receptors are expressed in key brain regions involved in feeding regulation, including the ARC, paraventricular nucleus (PVN), and DMH of the hypothalamus, as well as the area postrema (AP) of the medulla [31]. Furthermore, most GIPR agonists have been shown to reduce food intake in high-fat diet-induced obese mice [32]. When GIPRAs and GLP-1RAs were co-administered to obese mice, the combination significantly enhanced both appetite suppression and weight loss compared to monotherapy [33, 34]. These preclinical findings support clinical trial results showing that tirzepatide, a dual GIPR/GLP-1R agonist, exhibits greater anti-obesity effects than GLP-1 receptor agonists alone. Both GIPR and GLP-1R are expressed in overlapping brain regions including the ARC, PVN, and AP; however, they are localized to distinct neuronal populations [30, 35]. This anatomical distinction suggests that GIPRAs and GLP-1RAs act on different neuronal circuits, and their signals may be integrated within the brain, leading to synergistic anti-obesity effects.

A newly identified advantage of dual GIPR/GLP-1R agonists is their ability to reduce the adverse effects of nausea and vomiting, which are commonly associated with GLP-1RAs. In studies using the shrew, a well-established animal model for nausea and vomiting, co-administration of GIPRA almost completely abolished GLP-1RA-induced nausea while preserving its anorexigenic effect [32]. The proposed mechanism is that GIPRA inhibits GLP-1R-induced activation of the AP, a key brain region responsible for vomiting. Furthermore, this effect of GIPRA depends on GIPR expression in AP neurons [30].

### Clinical studies on the dual GIP/GLP-1 receptor agonist, tirzepatide

Tirzepatide has demonstrated significant weight loss effects in phase III clinical trials. The SURPASS program, which targeted patients with type 2 diabetes [29, 36–42], and the SURMOUNT program, which included individuals with obesity but without diabetes [43–45], both reported clinically significant reductions in body weight. Furthermore, tirzepatide has shown greater weight loss effects than the GLP-1RA semaglutide. In a clinical trial of patients with type 2 diabetes, weekly tirzepatide at 5, 10, or 15 mg for 40 weeks resulted in an 8.5–12.4% weight loss, compared to 6.7% with semaglutide at 1 mg/week [29].

In clinical trials, approximately 70% of participants reportedly exhibited adverse events, primarily gastrointestinal symptoms, such as diarrhea and nausea. The incidence of these side effects was comparable to that of semaglutide [37]. Animal studies suggest that GIPR signaling may help reduce nausea caused by GLP-1RA [32]. However, current clinical trials have not shown a significant difference in side effect rates between GIPR/GLP-1R dual agonist and GLP-1RA alone. Future studies are needed to further clarify the clinical advantages of GIPR/GLP-1R dual agonists beyond weight loss.

## Endogenous gut-derived GLP-1 and its anti-obesity and anti-diabetic effects

GLP-1 is a gut hormone synthesized and secreted by enteroendocrine L cells, which are widely distributed throughout the small intestine and colon. In addition to its peripheral production, GLP-1 is also synthesized in certain brain regions, primarily the nucleus tractus solitarius in the medulla [46]. The 180-residue pro-glucagon precursor undergoes proteolytic processing to generate active GLP-1, which exists in the forms of GLP-1(7-37) and GLP-1(7-36)amide. However, active GLP-1 is rapidly degraded by dipeptidyl peptidase IV, resulting in a remarkably short plasma half-life of approximately 2 min, with only 10–15% of the active hormone remaining in circulation [47]. Due to this rapid degradation, endogenous GLP-1 secreted from the gut must exert its biological effects immediately upon release. It has been postulated that specialized physiological systems exist to detect and respond to gut-derived GLP-1 in a timely manner. As research has progressed, it has been revealed that vagal sensory neurons distributed in the intestinal and hepatic portal regions play a crucial role in sensing circulating GLP-1 and mediating its physiological actions.

Vagal sensory nerves (vagal afferent nerves) are a type of visceral sensory nerves that connects peripheral organs to the brain. The cell bodies of vagal sensory neurons are clustered in the nodose ganglion, and recent transcriptomic profiling based on single-cell RNA sequencing analysis has identified more than a dozen genetically distinct subclasses [48, 49]. Among these subclasses, certain vagal sensory neurons express GLP-1Rs. Our ex vivo experiments using single cells isolated from the nodose ganglion revealed that approximately 10% of the neurons exhibited GLP-1-induced increases in cytosolic Ca^2+^ concentrations [50]. To investigate the physiological functions of endogenous GLP-1 mediated via vagal sensory neurons, a rat model with vagal sensory neuron-specific GLP-1R knockdown was generated by microinjecting a viral vector expressing GLP-1R shRNA into the nodose ganglion [51]. In these animals, both meal size and food intake during refeeding after fasting were increased, and postprandial insulin secretion was reduced, leading to elevated postprandial blood glucose levels [51]. These findings demonstrate that gut-derived GLP-1 secreted postprandially regulates feeding behavior and glucose metabolism via GLP-1Rs expressed on vagal sensory neurons.

It is well-known that GLP-1 is strongly secreted in response to the intake of macronutrients, including carbohydrates, proteins, and lipids. When considering the function of gut-derived GLP-1, it is difficult to ignore the metabolic effects of nutrient-derived energy in addition to the direct actions of GLP-1 itself. Recent studies have identified phytochemicals and polyphenols, which are non-caloric compounds, as novel factors that promote GLP-1 secretion [52]. We have also identified gastrointestinal distension stimuli [53] and the rare sugar d-allulose [54] as non-caloric stimulators of GLP-1 release. Furthermore, we demonstrated in animal studies that GLP-1 secretion induced by these factors does not evoke aversive behaviors, unlike GLP-1RAs, and promotes satiation, thereby suppressing hyperphagia and weight gain induced by a high-fat diet.

**Fig. 2:**
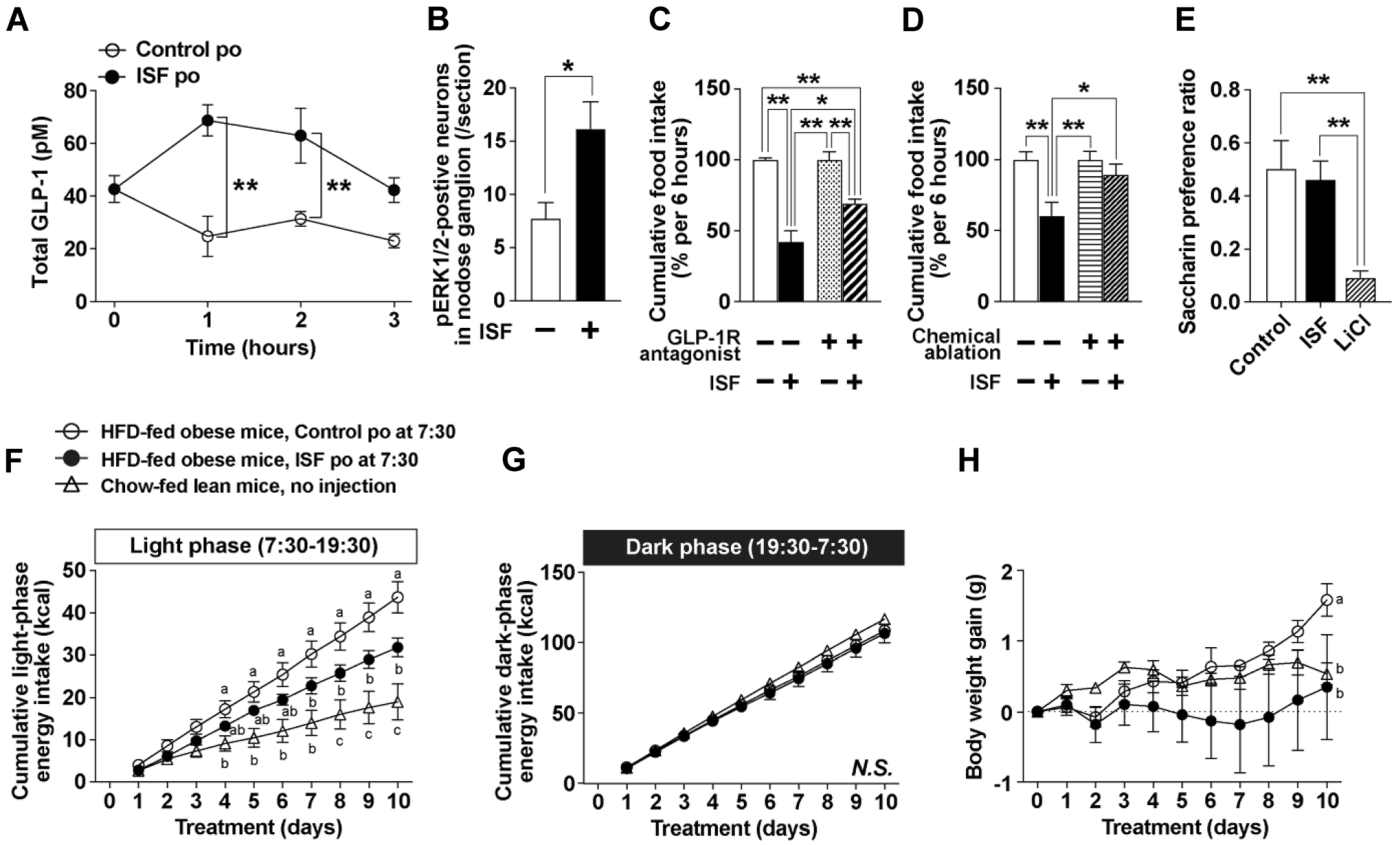
Anti-obesity effects mediated by GLP-1 through gastrointestinal distension induced by pectin-containing carbonated water (inflating stomach formulation, ISF). A single peroral administration of ISF at 30 ml/kg to C57BL/6J mice induces gastrointestinal distension, increases total GLP-1 concentration in the portal vein (**A**), and increases the number of pERK1/2-positive nodose ganglion neurons, a neural activation marker (**B**). ISF suppresses short-term food intake, and this effect is significantly attenuated by pretreatment with the GLP-1R antagonist exendin (9-39) (**C**) or by chemical ablation of vagal sensory nerves using capsaicin (**D**). In the conditioned taste aversion test, ISF does not induce aversion, unlike aversion-inducing lithium chloride (LiCl, **E**). Compared to chow-fed lean mice (open triangle), high-fat diet (HFD)-fed obese mice (open circle) exhibited a significant increase in food intake during the light phase (**F**) but not during the dark phase (**G**), thereby increasing body weight (**H**). Sub-chronic administration of ISF once daily at the light phase onset (filled circle) prevents light phase hyperphagia and body weight gain (**F–H**). In **A, F**, and **H**, **p < 0.01 or different letters indicate p < 0.05 by two-way ANOVA followed by Bonferroni’s test. In **B**, *p < 0.05 by unpaired t-test. In **C–E**, *p < 0.05 and **p < 0.01 by one-way ANOVA followed by Tukey’s test. All data are shown as means ± SEM. *n* = 4–8. Reprinted from Ohbayashi K, Iwasaki Y, et al. Front Endocrinol. 12:676869 (2021) [53] with slight modification

Consuming bulky foods with low energy density and high volume, such as salad, at the beginning of a meal induces satiation and attenuates postprandial increases in blood glucose levels [55–57]. This effect is thought to involve mechanical stimulation of visceral sensory nerves due to gastric distension, as well as delayed digestion and absorption caused by dietary fiber. We previously investigated how gastrointestinal distension regulates appetite and energy homeostasis using the inflating stomach formulation (ISF), a carbonated solution containing pectin that forms stable gel bubbles under acidic conditions in the stomach. ISF induced gastric and intestinal distension, which increased circulating GLP-1 levels and activated a subset of vagal sensory neurons (Fig. 2A, B). Gastrointestinal distension by ISF significantly reduced short-term food intake without aversive behavior, and this effect was significantly attenuated by pretreatment with GLP-1R antagonist or chemical ablation of vagal sensory neurons (Fig. 2C–E). Furthermore, subchronic administration of ISF suppressed light-phase hyperphagia in high-fat diet-induced obese mice and significantly inhibited body weight gain (Fig. 2F–H). These findings suggest that gastrointestinal distension serves as a physiological stimulus for GLP-1 secretion and that this mechanism may be effective in preventing and ameliorating hyperphagic obesity.

It is well-established that GLP-1 receptor agonists act on GLP-1 receptors expressed in pancreatic β-cells to enhance glucose-induced insulin secretion [46]. In contrast, whether the unstable gut-derived GLP-1 directly acts on pancreatic β-cells remains controversial [58]. Previous studies have demonstrated that gut-derived GLP-1 activates vagal sensory nerves, triggering insulin secretion via brain-autonomic nervous system pathways, a mechanism referred to as the "neuro-incretin effect" [51, 59]. Furthermore, our studies on ISF and the rare sugar d-allulose have shown that vagal sensory nerve activation induced by enhanced gut GLP-1 secretion potentiates insulin action, thereby improving glucose tolerance [53, 54]. This early GLP-1-mediated enhancement of insulin sensitivity represents a novel physiological function of GLP-1. To fully elucidate the physiological roles of gut-derived GLP-1, further investigation is needed into the function of GLP-1 receptor-expressing vagal sensory nerves. Moreover, gut GLP-1 may have additional physiological functions beyond the regulation of feeding behavior and glucose metabolism.

## Conclusion

**Fig. 3:**
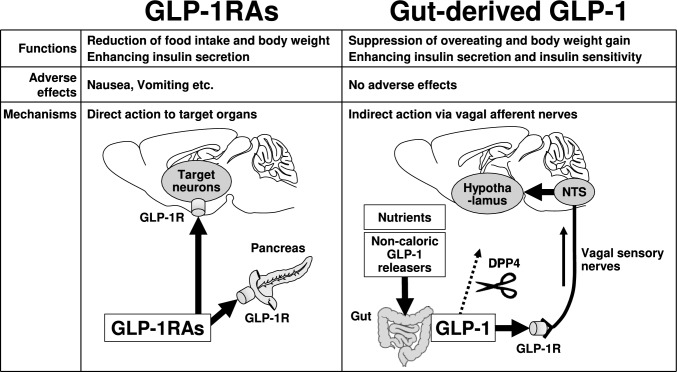
Comparison of functions and mechanisms of GLP-1RAs vs. gut-derived GLP-1. GLP-1RAs exert anti-obesity and anti-diabetic effects by directly acting on GLP-1 receptors in target organs. However, GLP-1RAs cause adverse effects such as nausea and vomiting by binding to GLP-1 receptors on neurons beyond those involved in feeding regulation. In contrast, gut-derived GLP-1, which is secreted in response to nutrients or non-caloric GLP-1 releasers, such as gastrointestinal distension [53] and d-allulose [54], acts on GLP-1Rs in vagal sensory nerves. The neural signals from the vagal sensory neurons activated by gut GLP-1 are likely transmitted to limited brain regions, thereby preventing hyperphagic obesity and improving glucose tolerance by enhancing insulin action without causing side effects

Endogenous gut-derived GLP-1 and GLP-1RAs are both agonists of the GLP-1R; however, they act on different target organs and thus exert distinct physiological/pharmacologic effects. GLP-1RAs act on GLP-1Rs expressed in the brain and pancreatic β-cells, regulating feeding behavior and insulin secretion. Furthermore, GLP-1RAs can also bind to GLP-1Rs on neurons beyond those involved in feeding regulation, leading to adverse effects, such as nausea and vomiting (Fig. 3). In contrast, gut-derived GLP-1 primarily acts on GLP-1 receptors expressed on vagal sensory nerve terminals located near the intestines. The neural signals generated by this activation are selectively transmitted to specific brain regions, enabling the regulation of feeding behavior and glucose metabolism (insulin secretion and insulin action) without inducing adverse effects (Fig. 3). The secretion of gut-derived GLP-1 is significantly influenced by meal composition and meal sequence [60]. In an intervention study on meal sequence, individuals with prediabetes were instructed to consume GLP-1-stimulating foods, such as meat, fish, or salad, at the beginning of a meal, followed by a 5-min waiting period before consuming carbohydrate-rich foods, such as rice or bread. As a result, this approach was reported to not only improve glycemic control but also result in greater weight loss [61]. A major concern with GLP-1RAs, despite their potent anti-obesity effects, is post-treatment weight rebound [16]. This rebound is likely driven by increased food intake following drug discontinuation. The effects of gut-derived GLP-1 secretion are considered to be more significant in preventing weight gain rather than directly inducing weight loss, based on findings from animal studies on GLP-1 releasers including gastrointestinal distention [53] and d-allulose [54]. In the future, the combined use of GLP-1RAs and GLP-1 releasers, which have distinct mechanisms of action and effects, may contribute to the development of a long-term and effective strategy for obesity treatment and prevention.
